# Supplementary material for: Single crystal of a one-dimensional metallo-covalent organic framework
Source: Nat Commun. 2020 Mar 18;11:1434. doi: 10.1038/s41467-020-15281-1 (PMC7080745; doi:10.1038/s41467-020-15281-1)
Supplement: Supplementary file 1 — Supplementary Information [file 41467_2020_15281_MOESM1_ESM.pdf]

Supplementary Information

Single crystal of a one-dimensional metallo-covalent organic framework

Xu et al.

This PDF file includes:

Supplementary Methods
Supplementary Notes
Supplementary Figures 1-31
Supplementary Tables 1-2
Supplementary References

Supplementary Methods

Reagent

All commercially available starting compounds and solvents were purchased from commercial sources and used without further purification. 2-(4-(dimethoxymethyl)phenyl)-4,4,5,5-tetramethyl-1,3,2-dioxaborolane was synthesized through a two-step reaction as reported in literature[1].

Instrumentation and characterization

Liquid ^{1}H and ^{13}C NMR spectra were recorded on a Bruker AVANCE I 300 MHz or a Bruker AVANCE I 500 MHz NMR spectrometer and chemical shifts (δ-scale) were reported in parts per million (ppm). Solid-state NMR experiments were performed on a Bruker Avance III HD 600 MHz wide-bore NMR spectrometer. The ^{13}C cross-polarization magic-angle spinning (CP/MAS) NMR spectra were recorded with a 4 mm double-resonance MAS probe. High-resolution mass spectrometry (HRMS) data were obtained on a Finnigan/MAT 95XL-T spectrometer. Fourier-transform infrared spectroscopy (FTIR) was carried out on a Bruker VERTEX 80v spectrometer in transmission mode under vacuum. Photoluminescence (PL) spectra were obtained with a laser confocal microscope (NT-MDT, NTEGR Spectra) with 532 nm (continuous wave (cw)) excitation lasers under ambient conditions. H, C and N contents were determined *via* elemental analysis performed on an Elementar vario MICRO cube. Thermogravimetric analysis (TGA) was carried out on a Discovery TGA within the temperature range of 30 °C to 800 °C with a heating rate of 10 °C/min.

Powder X-ray diffraction (PXRD) data were collected on a Bruker D8 Focus Powder X-ray Diffractometer using Cu K_{α} radiation (40 kV, 40 mA) at room temperature. Synchrotron powder X-ray diffraction (SPXD) data were collected on the sample in a 0.5 mm capillary on the BL14B1 beamline (λ = 0.69005 Å) at the Shanghai Synchrotron Radiation Facility in Shanghai, China. The collected data were ranging from 2.0 to 20.0°, with 0.004° data binning.

Nitrogen sorption analysis was performed on Quantachrome Instruments Autosorb-iQ (Boynton Beach, Florida USA) with extra-high pure gases.

Scanning electron microscopy (SEM) images were obtained with a JEOL JSM-6701F microscope. A JEM-2100Plus (LaB6 filament) operated at 200 kV was used for high resolution transmission electron microscopy (HRTEM) imaging. Single-crystal electron diffraction (SCED) data were collected using a JEOL JEM2100 TEM (LaB6 filament) operating at 200 kV. The reciprocal space reconstruction was carried out using the RED software, and the reflection intensity extraction was conducted by the program XDS[2,3].

For the second harmonic generation (SHG) characterization, a Yb:KGW femtosecond PHAROS laser system was used as the pump of a collinear optical parametric amplifier ORPHEUS with a LYRA wavelength extension option (Light Conversion Ltd, pulse duration of 150 fs, tunable excitation wavelength of 310 nm – 2700 nm, and repetition rate of 100 Hz –100 kHz). The laser

beam was focused on the COF samples with a 100x (NA = 0.9) air objective from Nikon (1 μm^2 spot size). The nonlinear emission was collected in a back-scattering configuration *via* the same objective and detected by a spectrograph (PI Acton SP2300 by Princeton Instruments) for spectral measurements. The power of the collected SHG emission was measured using a calibrated silicon photodetector (Newport), while the excitation power was measured using a germanium photodetector (Thorlabs). For polarization dependent SHG measurements, a half-wave plate was used to vary the orientation of the linearly polarized laser pulses.

X-ray photoelectron spectroscopy (XPS) was performed using PHI Quantera II with a monochromatic Al-Kα source (energy = 1486.6 eV).

Atomic force microscopy (AFM) nano-indentation experiments were performed using the Dimension Icon instrument operating under the indentation mode, equipped with a Bruker Tap525A probe. Its spring constant and contact sensitivity have been calibrated as 156.760 N/m and 83.01 nm/volt, respectively.

Supplementary Notes

Synthesis of 4,4'-(1,10-phenanthroline-2,9-diyl)dianiline (**I**)

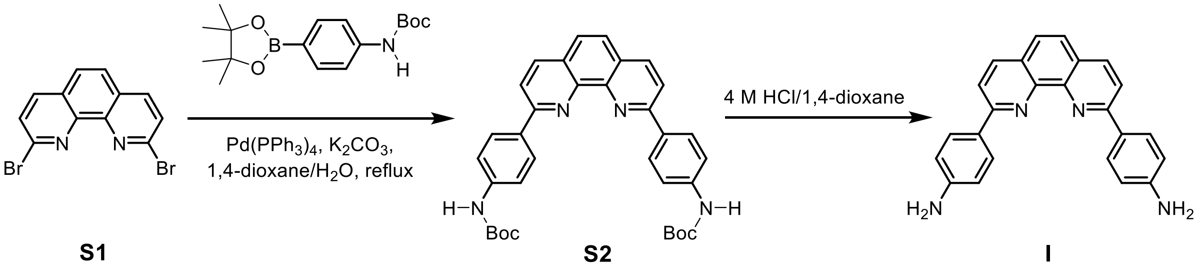

Compound S2. To a two-necked round bottom flask were added S1 (1.014 g, 3.0 mmol), *tert*-butyl (4-(4,4,5,5-tetramethyl-1,3,2-dioxaborolan-2-yl)phenyl)carbamate (2.490 g, 7.8 mmol), K_2CO_3 (1.242 g, 9.0 mmol), $Pd(PPh_3)_4$ (347 mg, 0.3 mmol), 1,4-dioxane (48 mL), and H_2O (12 mL). The reaction mixture was deaerated by slow bubbling of N_2 for 30 min, and then heated to reflux at 100 °C for 8 h. After cooling to room temperature, the solvent was removed under reduced pressure, the residue was treated with H_2O (60 mL), extracted with CH_2Cl_2 (4 × 40 mL), the combined organic phase was washed with brine, dried over Na_2SO_4, and concentrated. The crude product was purified by silica gel column chromatography using CH_2Cl_2/CH_3OH = 100/0.5 as the eluent to give the product **S2** (1.204 g, 71% yield) as a white solid. ^1H NMR (300 MHz, $CDCl_3$): δ = 8.48-8.39 (m, 4H), 8.26 (d, J = 8.5 Hz, 2H), 8.10 (d, J = 8.5 Hz, 2H), 7.75 (s, 2H), 7.64-7.53 (m, 4H), 6.65 (s, 2H), 1.57 (s, 18H). ^{13}C NMR (125 MHz, $CDCl_3$): δ = 156.1, 152.5, 139.7, 136.8, 134.1, 128.4, 127.7, 125.7, 119.4, 118.4, 80.8, 28.4. HRMS: m/z calcd for $C_{34}H_{35}N_4O_4$ $[M + H]^+$: 563.2653, found: 563.2664.

4,4'-(1,10-phenanthroline-2,9-diyl)dianiline (I). To a round bottom flask were added S2 (562.3 mg, 1.0 mmol) and 4 M HCl/1,4-dioxane (10 mL). The mixture was stirred at room temperature for 2 h, and then filtered to get a dark red solid. The solid was dissolved in deionized water (50 mL), and then neutralized with KOH solution. The resulting suspension was extracted with CH_2Cl_2 (3 × 40 mL), the combined organic phase was washed with brine, dried over Na_2SO_4, and concentrated to afford the product **I** (335 mg, 92% yield) as a light yellow solid. ^1H NMR (300 MHz, DMSO-d_6): δ = 8.36 (d, J = 8.5 Hz, 2H), 8.24 (d, J = 8.6 Hz, 4H), 8.16 (d, J = 8.5 Hz, 2H), 7.81 (s, 2H), 6.77 (d, J = 8.6 Hz, 4H), 5.57 (s, 4H). ^{13}C NMR (125 MHz, DMSO-d_6): δ = 155.6, 150.4, 145.2, 136.5, 128.3, 126.7, 126.3, 124.9, 118.3, 113.8. HRMS: m/z calcd for $C_{24}H_{19}N_4$ $[M + H]^+$: 363.1604, found: 363.1611.

Synthesis of 2,9-bis(4-(dimethoxymethyl)phenyl)-1,10-phenanthroline (**II**)

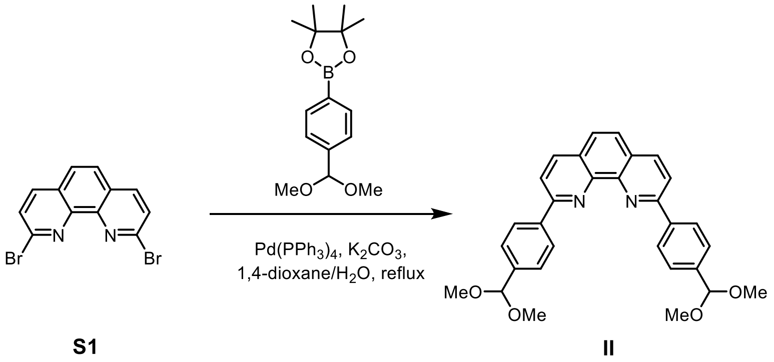

2,9-bis(4-(dimethoxymethyl)phenyl)-1,10-phenanthroline (II). To a two-necked round bottom flask were added S1 (676 mg, 2.0 mmol), 2-(4-(dimethoxymethyl)phenyl)-4,4,5,5-tetramethyl-1,3,2-dioxaborolane (1.45 g, 5.2 mmol), K_2CO_3 (828 mg, 6.0 mmol), $Pd(PPh_3)_4$ (231 mg, 0.2 mmol), 1,4-dioxane (32 mL), and H_2O (8 mL). The reaction mixture was deaerated by slowly bubbling N_2 for 30 min, and then heated to reflux at 100 °C for 8 h. After cooling to room temperature, the solvent was removed under reduced pressure. The residue was treated with H_2O (60 mL), extracted with CH_2Cl_2 (4 × 40 mL), the combined organic phase was washed with brine, dried over Na_2SO_4, and concentrated. The crude product was purified by silica gel column chromatography using hexane/ethyl acetate = 3/1 as the eluent to give the product **II** (758 mg, 79% yield) as a white solid. ^{1}H NMR (300 MHz, $CDCl_3$): δ = 8.51-8.40 (m, 4H), 8.33 (d, J = 8.4 Hz, 2H), 8.16 (d, J = 8.4 Hz, 2H), 7.81 (s, 2H), 7.68 (d, J = 8.3 Hz, 5H), 5.54 (s, 3H), 3.39 (s, 11H). ^{13}C NMR (125 MHz, $CDCl_3$): δ = 156.5, 146.1, 139.6, 139.2, 136.9, 127.9, 127.6, 127.3, 126.0, 120.1, 102.8, 52.5. HRMS: m/z calcd for $C_{30}H_{29}N_2O_4$ $[M + H]^+$: 481.2122, found: 481.2131.

Synthesis of the conjugated polymer

I (11.6 mg, 0.032 mmol) and **II** (15.4 mg, 0.032 mmol) were weighed into a 10 mL Schlenk tube. To the mixture were added 1-butanol (0.1 mL), 1,2-dichlorobenzene (0.9 mL) and 6 M aqueous acetic (0.1 mL). The Schlenk tube was frozen in a liquid nitrogen bath, evacuated to an internal pressure of 0 mbar and sealed. After warming to room temperature, the Schlenk tube was placed into an oven and heated at 120 °C for 3 days yielding a yellow-orange solid at the bottom of the tube. The Schlenk tube was opened when the mixture was still warm and the solid was transferred into a vial, separated by centrifugation, washed copiously with DMSO, THF, and EtOH. After drying at room temperature, the resulting solid was dried under vacuum at 100 °C for 12 h to obtain the conjugated polymer.

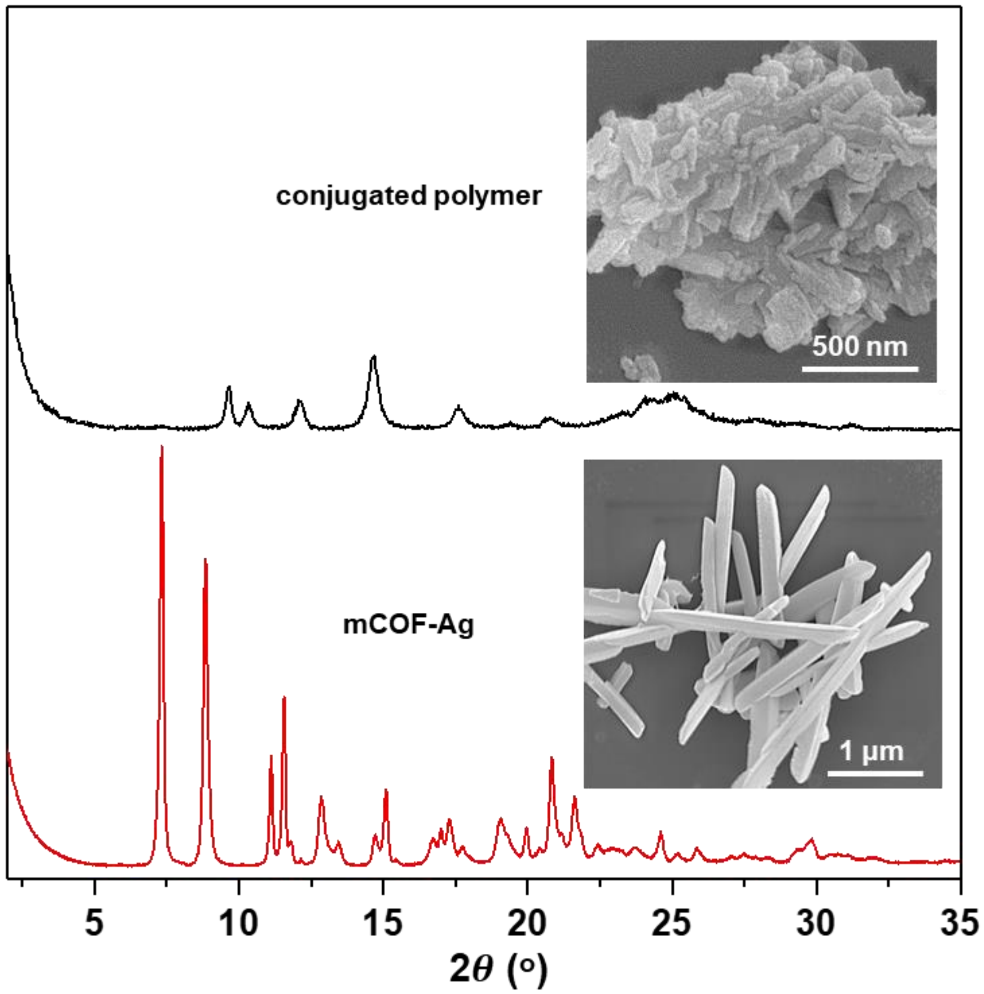

Supplementary Figure 1 | Experimental PXRD patterns of the conjugated polymer (black) and mCOF-Ag (red). The synthesised conjugated polymer has a semi-crystalline feature, which is attributed to the π-π stacking in the rigid and conjugated structure of phenanthroline backbones; besides, the reversibility in imine bond formation can impart self-correction during the hydrothermal conditions.

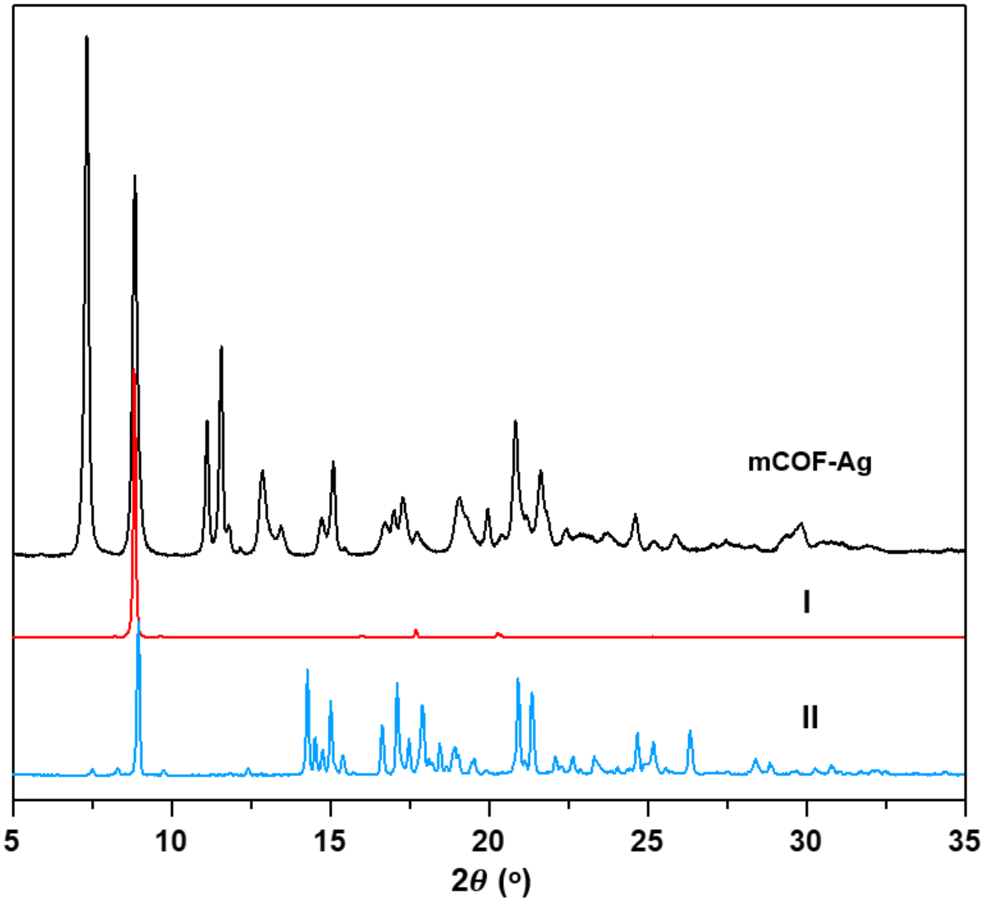

Supplementary Figure 2 | Experimental PXRD patterns of mCOF-Ag (black), building block **I** (red), and building block **II** (blue).

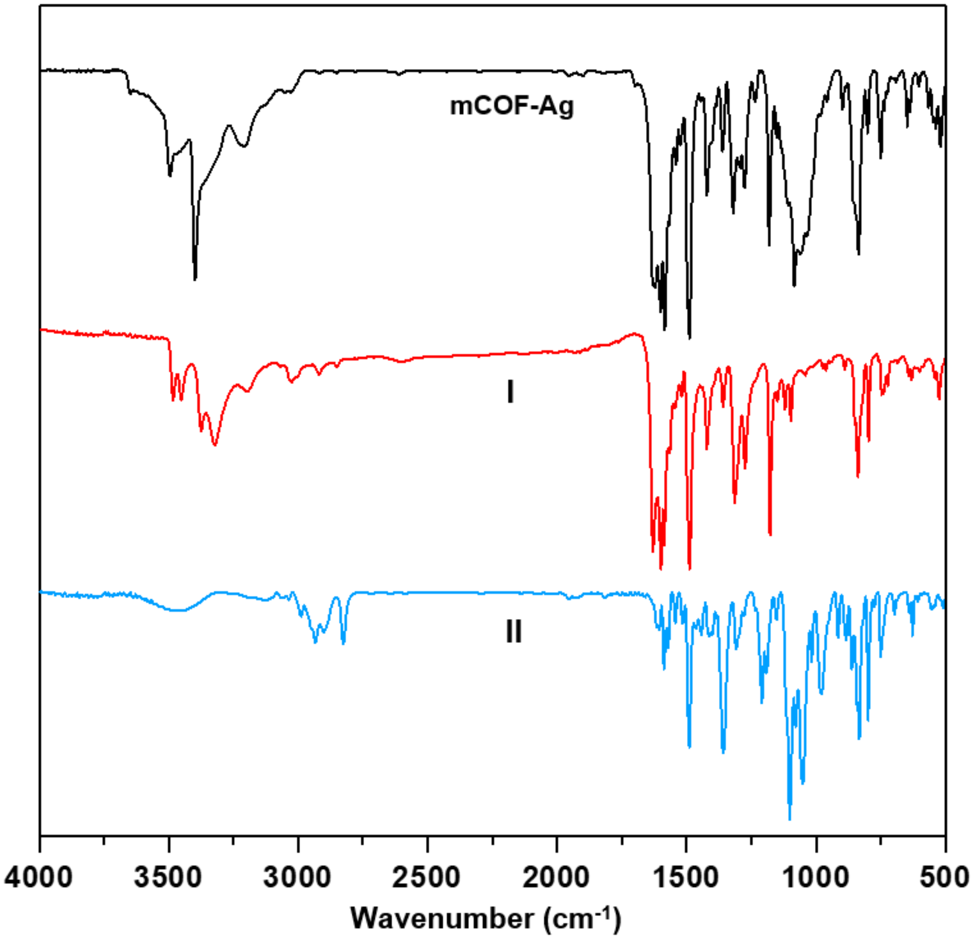

Supplementary Figure 3 | FT-IR spectra of mCOF-Ag (black), building block **I** (red), and building block **II** (blue).

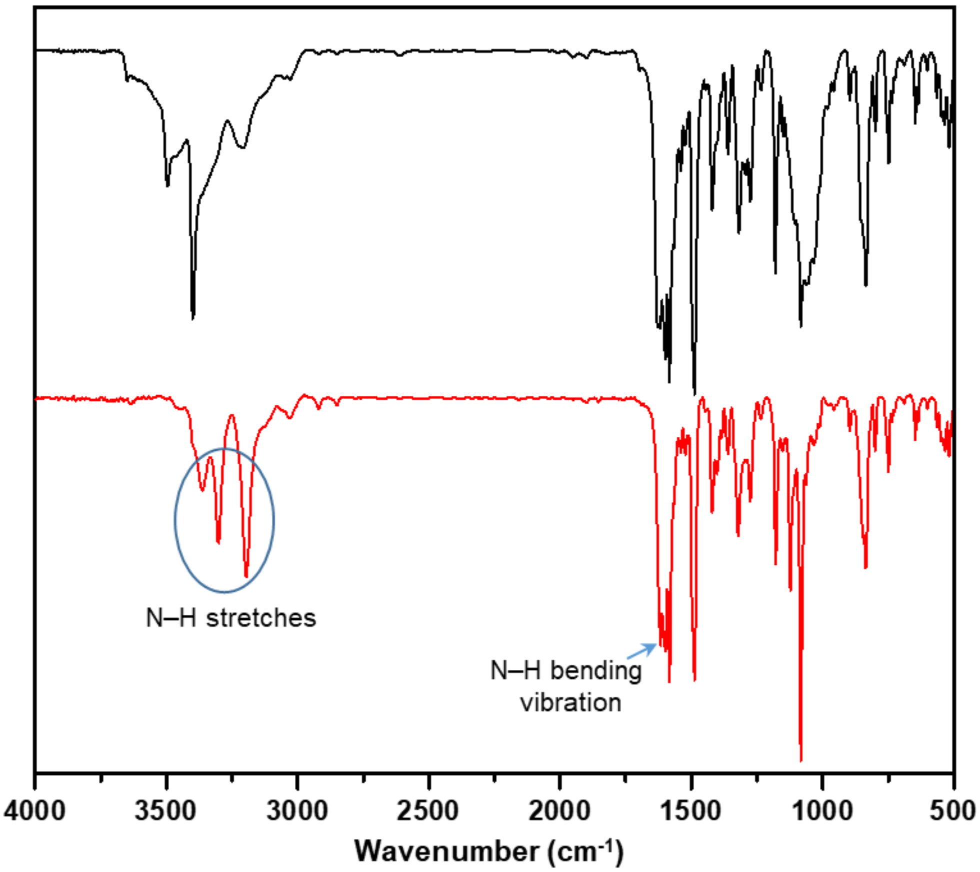

Supplementary Figure 4 | FT-IR spectra of mCOF-Ag before (black) and after (red) drying the KBr pellet at 100 °C to eliminate the influence of water. In the red curve, the characteristic peaks of N–H stretches and N–H bending vibration are apparent.

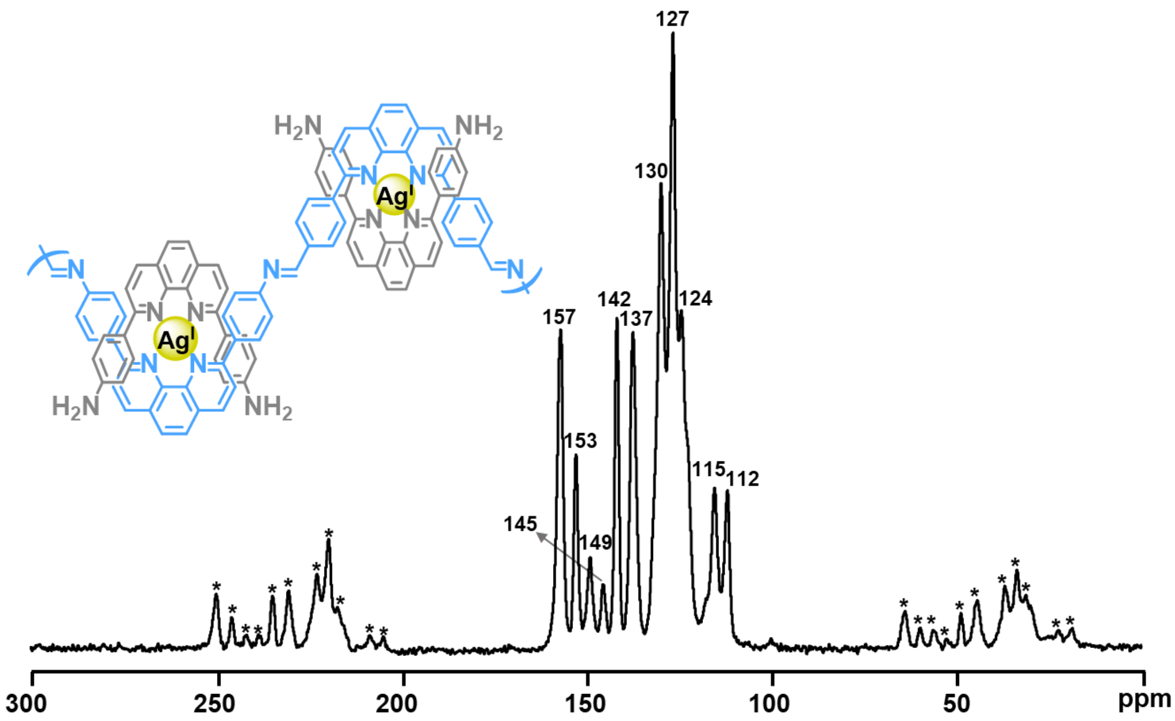

Supplementary Figure 5 | ^{13}C CP/MAS NMR spectra of mCOF-Ag. The asterisks denote the spinning sidebands.

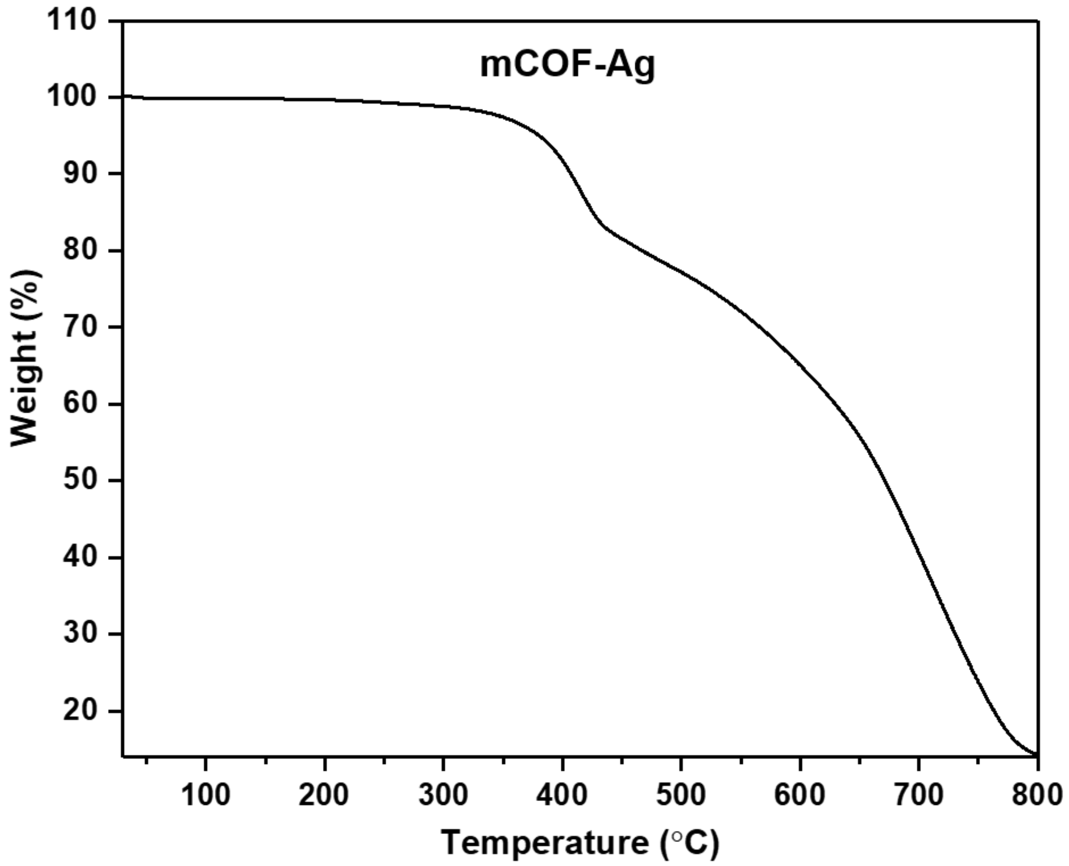

Supplementary Figure 6 | TGA curve of mCOF-Ag. The decomposition temperature is about 320 ºC. The ~1.4% weight loss below 320 °C is attributed to the small amount of solvents trapped in the crystal.

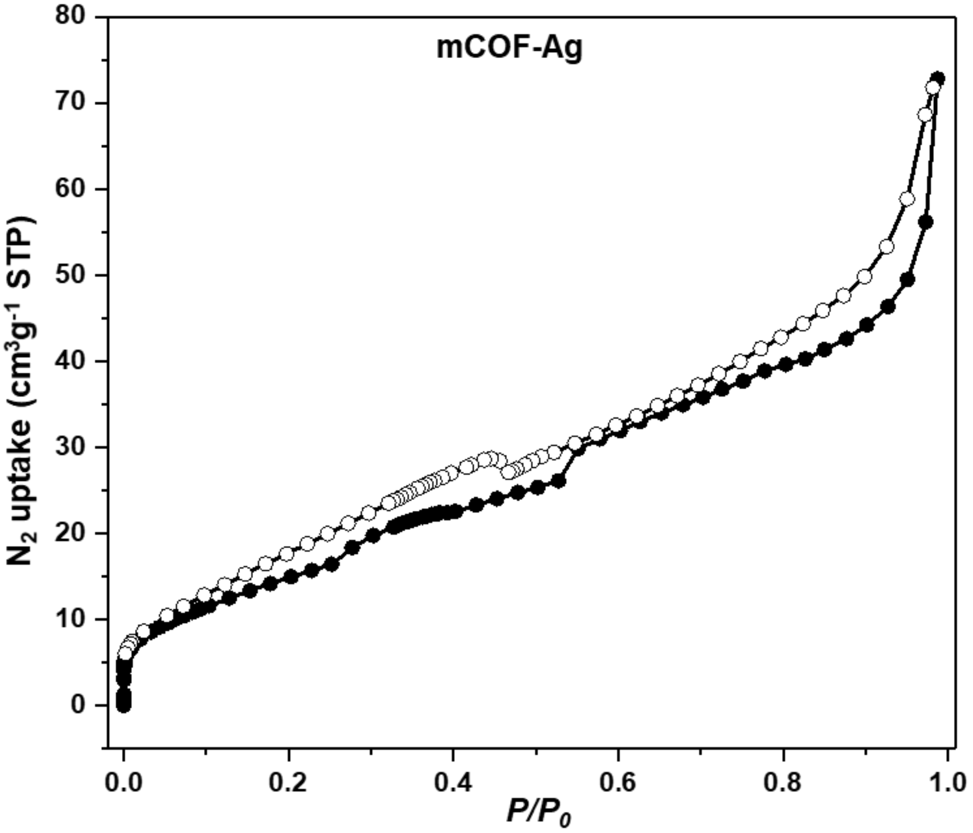

Supplementary Figure 7 | Nitrogen adsorption (filled symbols) and desorption (empty symbols) isotherms of mCOF-Ag. The low uptake in the isotherm line is in line with its crystal structure, whereby the pores are occupied by BF_4^- anions.

Supplementary Table 1 | SCED: Experimental and Crystallographic parameters of mCOF-Ag.

Tilt range (°)	-41.40 ~ 39.96
Tilt step (°)	0.23
Wave length (Å)	0.0251
No. of frames	336
Exposure time per image (s)	0.5
Crystal system	Monoclinic
Possible space group	*C*2/*c*, *Cc*, *C*2/*m*, *Cm*, *C*2
Unit cell parameters	a = 15.66 Å, b = 31.00 Å, c = 10.87 Å,<br>α = 90°, β = 123.31°, γ = 90°
Resolution (Å)	0.95
$I/\sigma(I)$	4.90
Completeness (%)	56
$CC_{1/2}$	98.8
R_{meas} (%)	11.8
No. of total reflections	4024
No. of unique reflections	1522

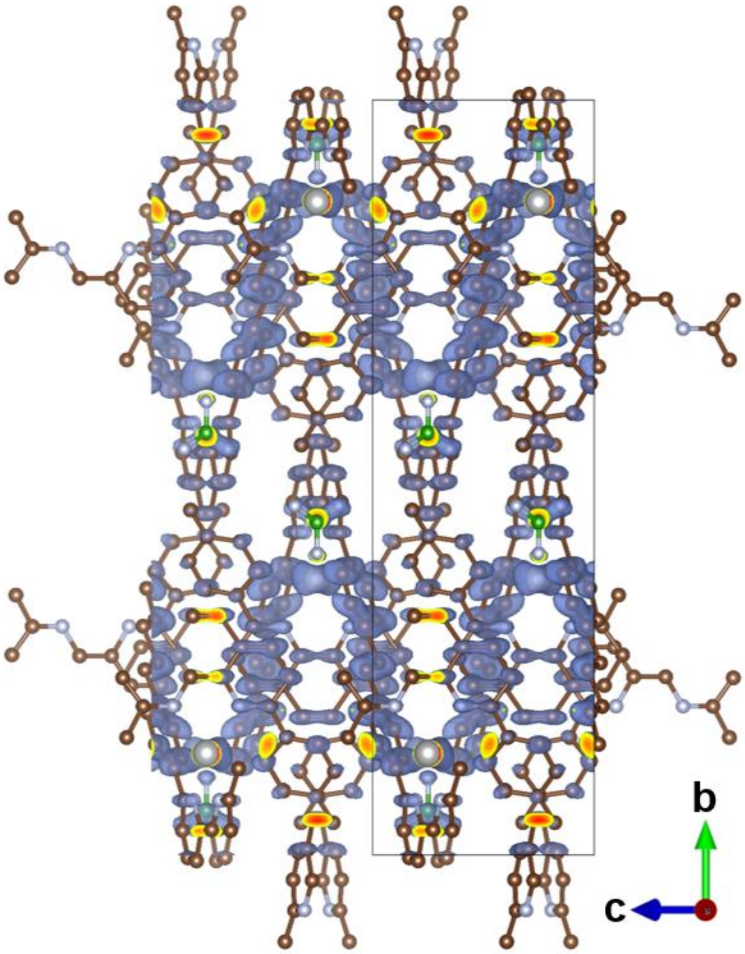

Supplementary Figure 8 | Observed electron density map of the structure of mCOF-Ag determined from SCED data along the *a*-axis. All positions of the non-hydrogen atoms (C, N, and Ag) on the framework and the locations of the guests ($BF4^{-}$ anions) are resolved.

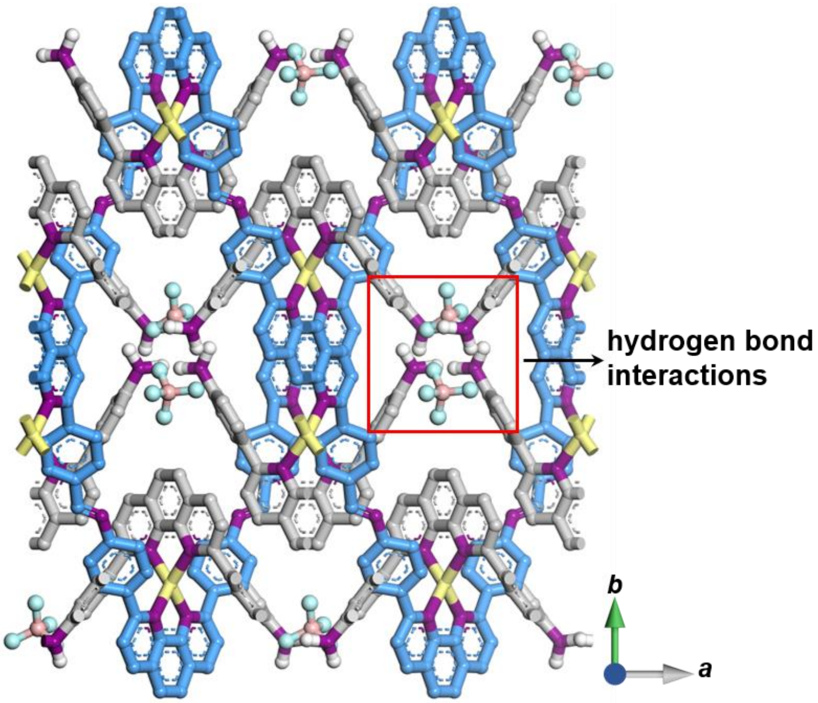

Supplementary Figure 9 | Crystal view along the *c*-axis, in which the H···F hydrogen bond interactions between amine and BF4⁻ anions are highlighted with a red square. Colour scheme: C on organic chains, blue; C on pendants, grey; N, purple; Ag, yellow; B, pink; F, green; H of amine groups, white.

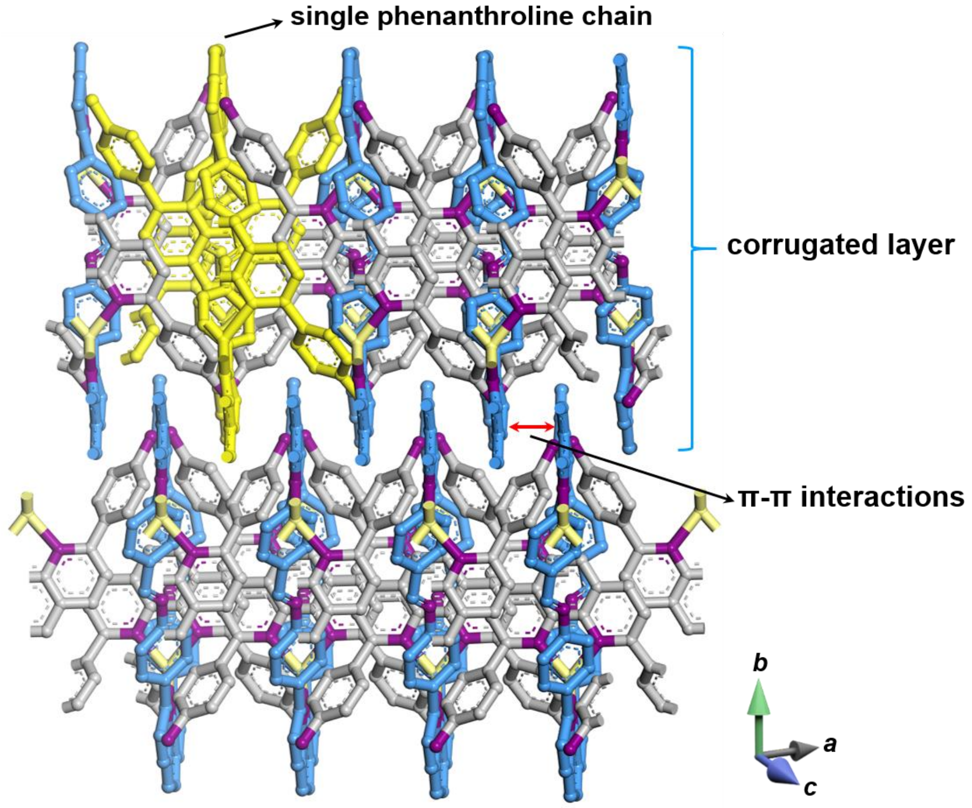

Supplementary Figure 10 | Tilted side view of the crystal structure, in which a single phenanthroline chain is highlighted in bright yellow. The corrugated layer and the π-π stacking of interlayer phenanthroline rings are also identified. C on organic chains, blue; C on pendants, grey; N, purple; Ag, yellow. Hydrogen atoms and $BF4^{-}$ anions are omitted for clarity.

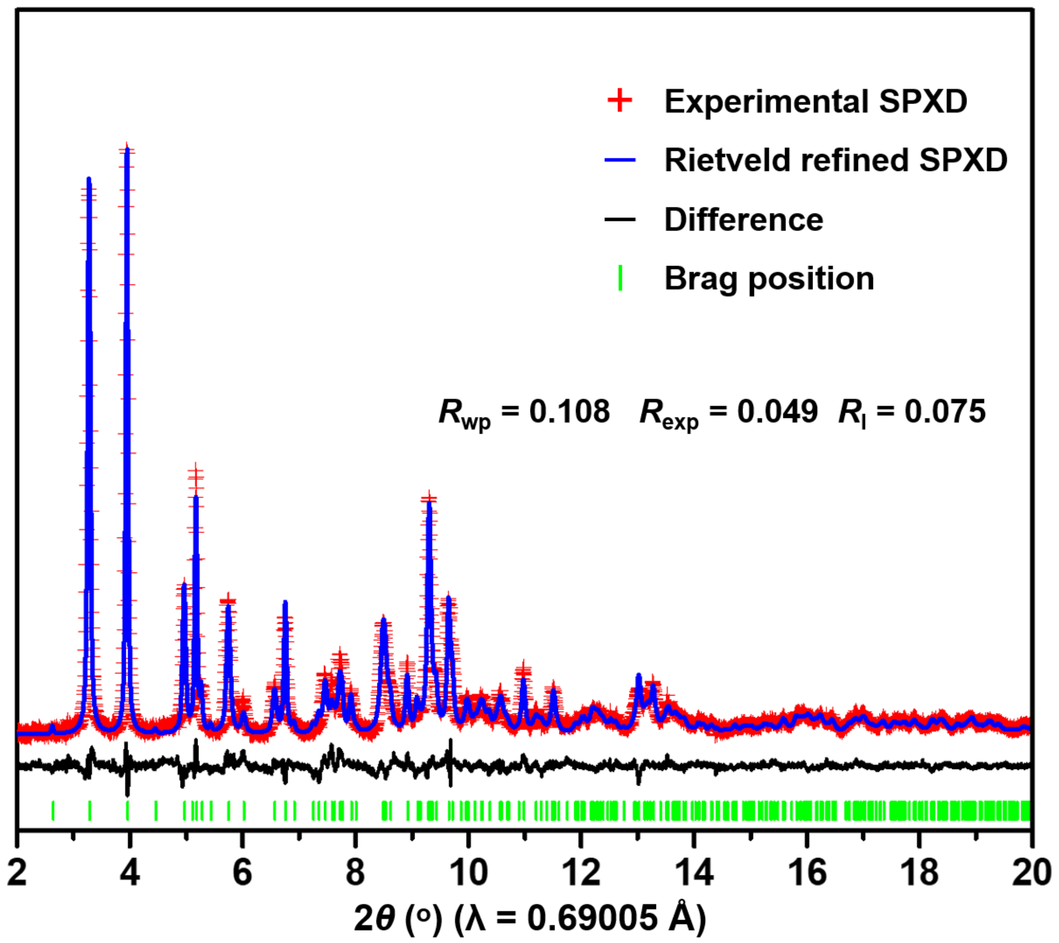

Supplementary Figure 11 | Structure refinement of mCOF-Ag against the SPXD data (Rietveld refinement). Experimental (red), Rietveld refined (blue), as well as difference profiles (black) are presented. The positions of Bragg reflections under the patterns are shown in green.

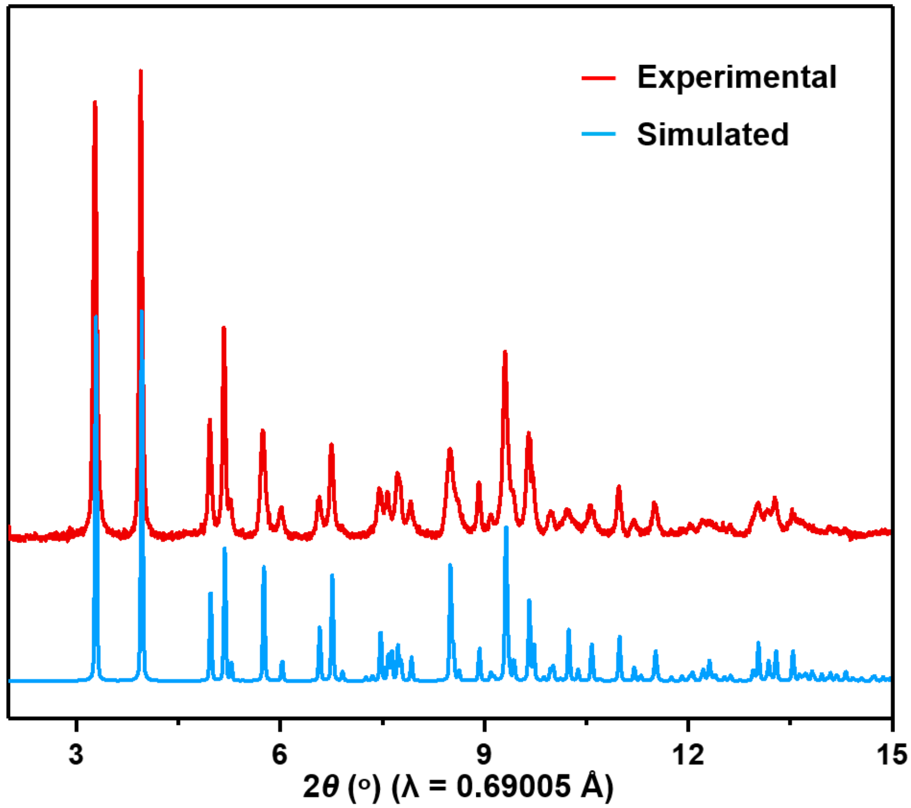

Supplementary Figure 12 | Experimental SPXD pattern and the simulated profile from the single crystal structure of mCOF-Ag. The simulated data is highly consistent with the experimental data, indicating the very high quality of the synthesized crystals.

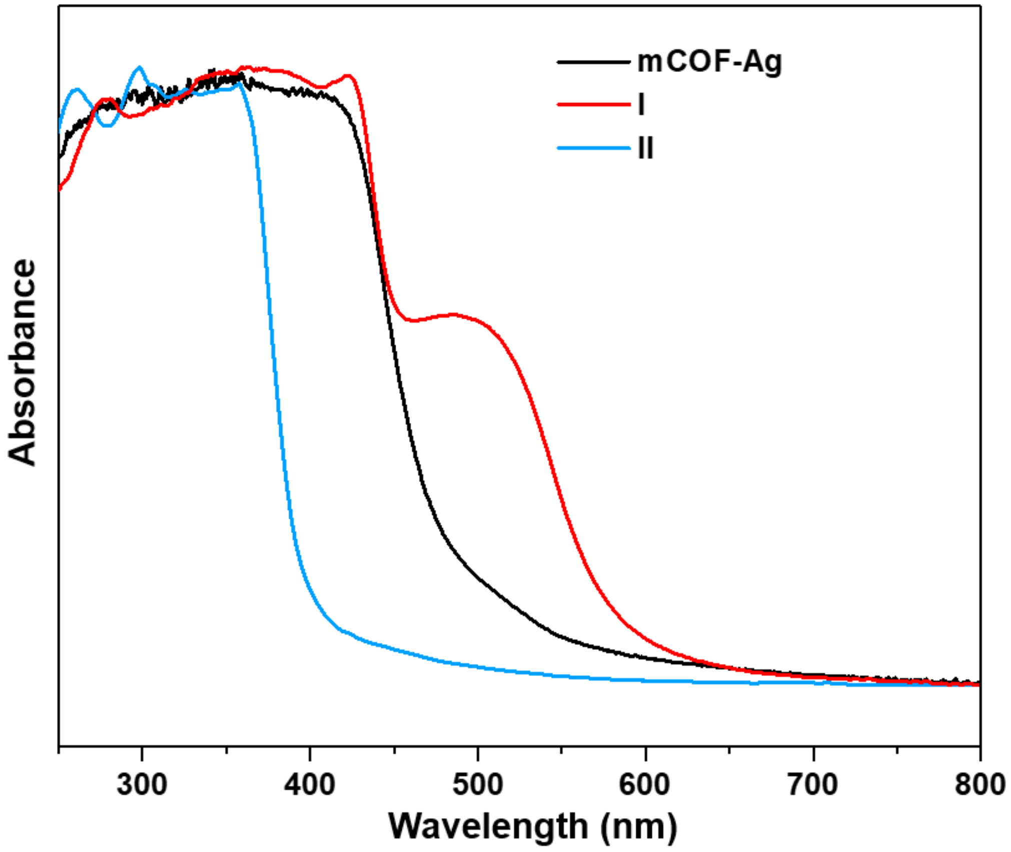

Supplementary Figure 13 | Uv-vis spectra of mCOF-Ag, building block **I**, and building block **II**.

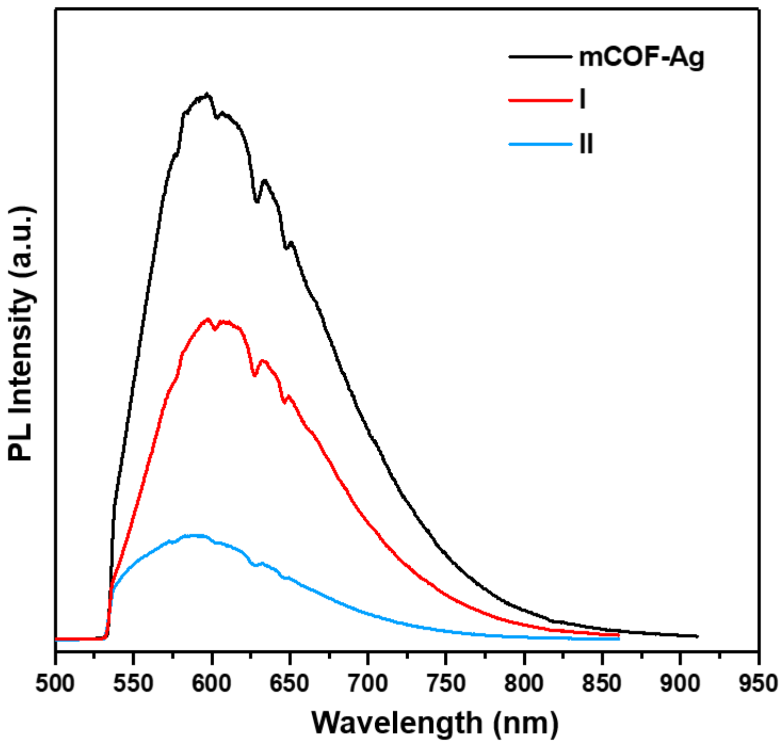

Supplementary Figure 14 | PL spectra of mCOF-Ag, building block **I**, and building block **II** excited by 532 nm laser.

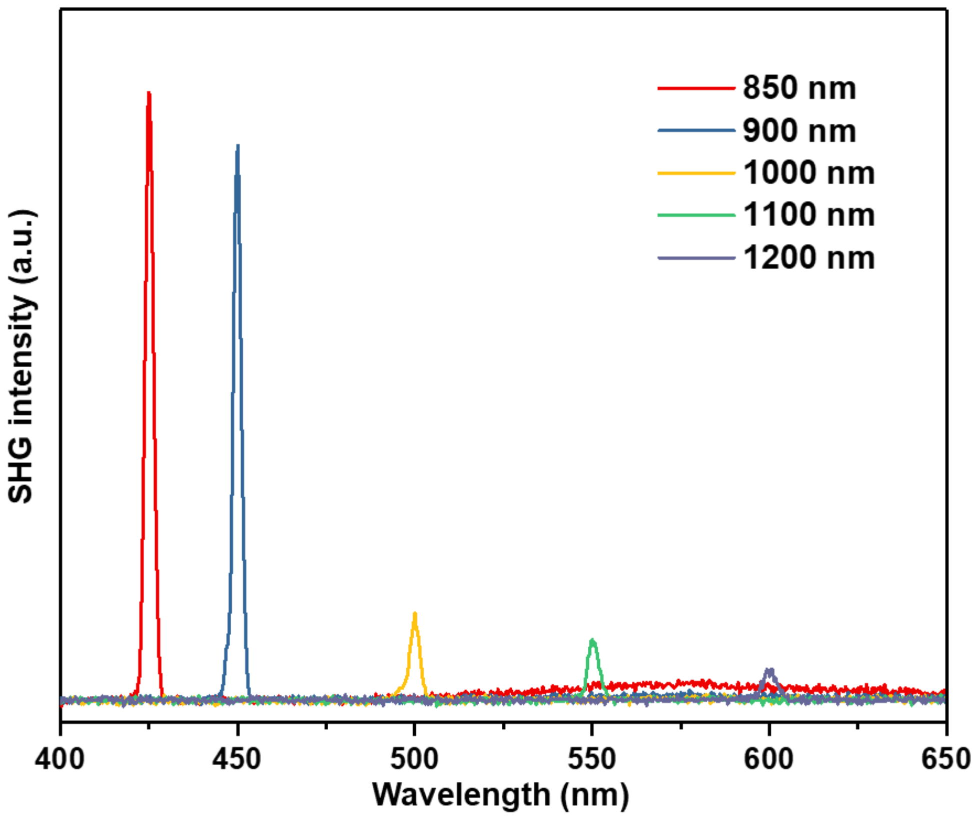

Supplementary Figure 15 | SHG spectra of an isolated mCOF-Ag crystal at different pump wavelengths.

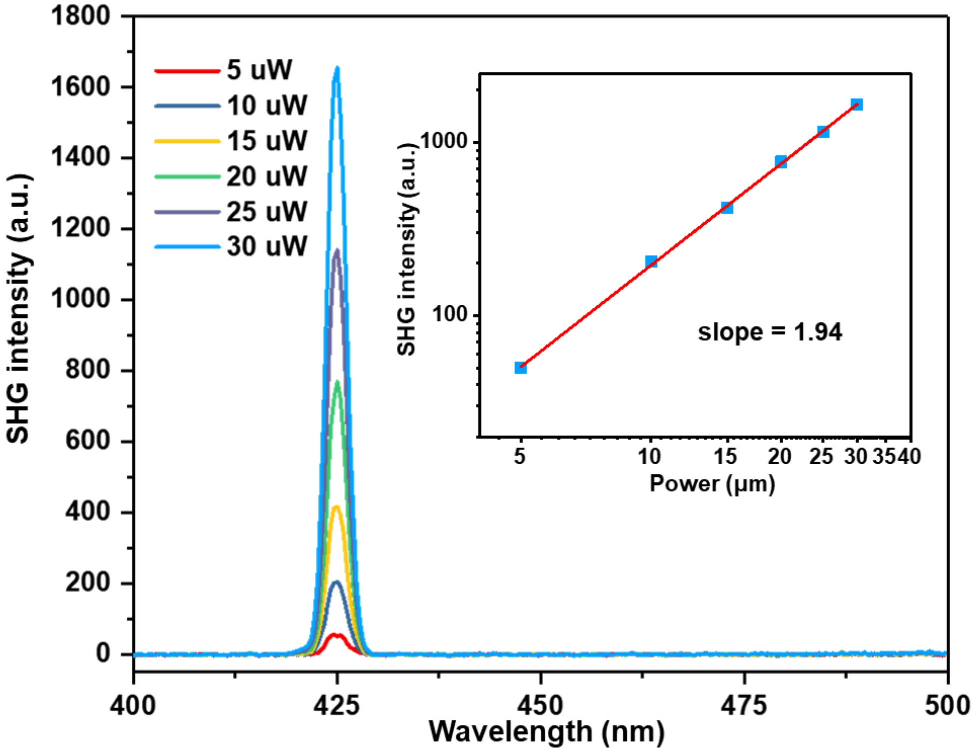

Supplementary Figure 16 | Pump power-dependent SHG spectra of an isolated mCOF-Ag crystal at 850 nm. Inset shows the linear relationship between SHG intensities and pump power, the fitting curve (red line) has a slope of 1.94, confirming the second-order nature of the nonlinear process.

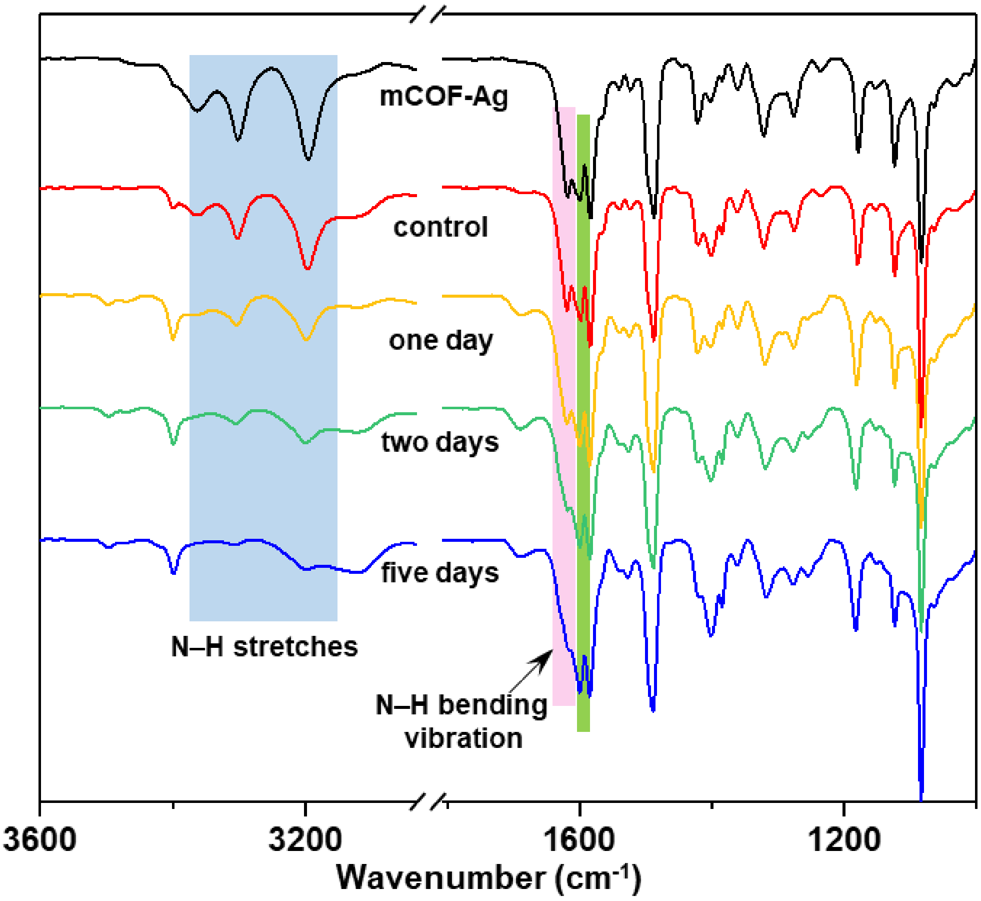

Supplementary Figure 17 | FT-IR spectra showing time evolution of vibrational bands when mCOF-Ag was cross-linked with glyoxal. With longer reaction time, the characteristic peaks of N−H stretches (blue band) and N−H bending vibration (pink band) decrease, indicating the consumption of amine groups, while the peak at 1600 cm^{-1} (green band) due to newly formed imine bond increases gradually. The red curve corresponds to the control experiment without glyoxal, which was heated in 1,4-dioxane at 70 °C for five days. All KBr pellets were dried at 100 °C before testing to eliminate background signals due to water.

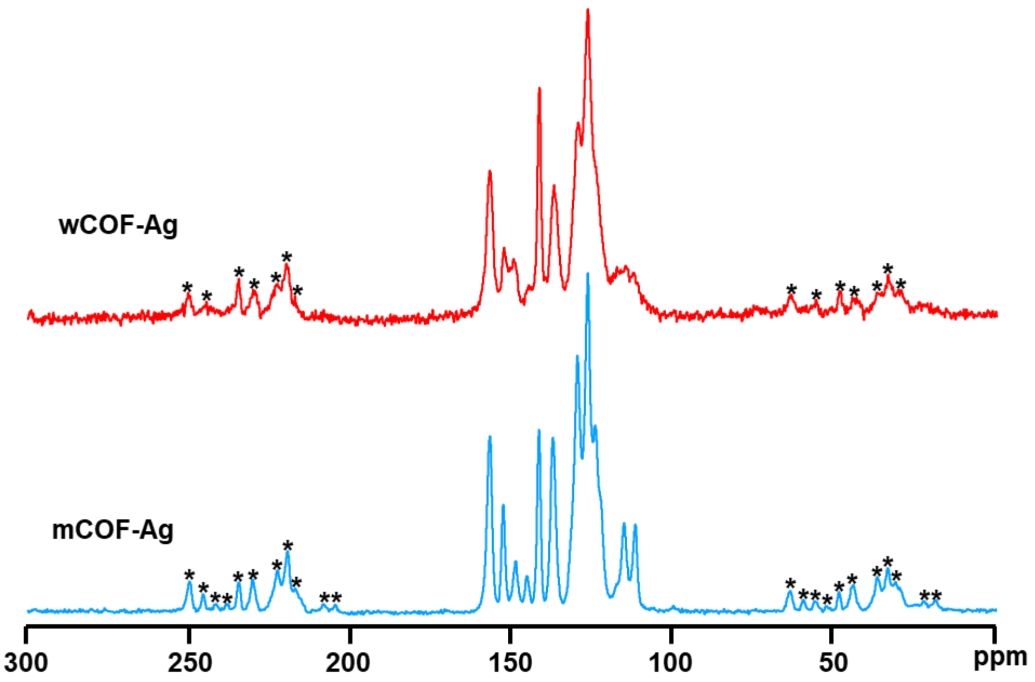

Supplementary Figure 18 | ^{13}C CP/MAS NMR spectra of mCOF-Ag and wCOF-Ag. The asterisks denote the spinning sidebands.

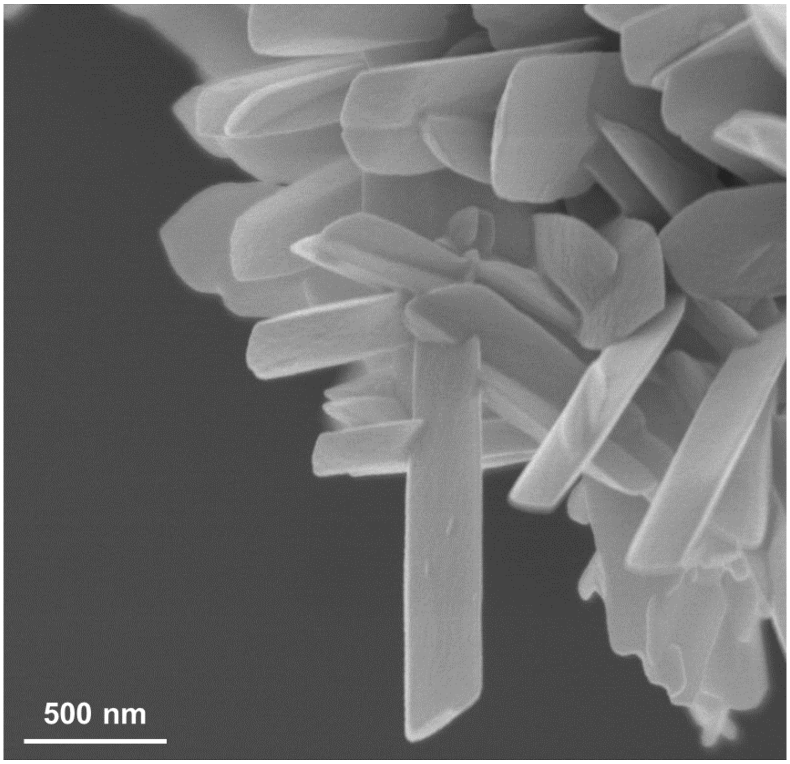

Supplementary Figure 19 | SEM image of wCOF-Ag.

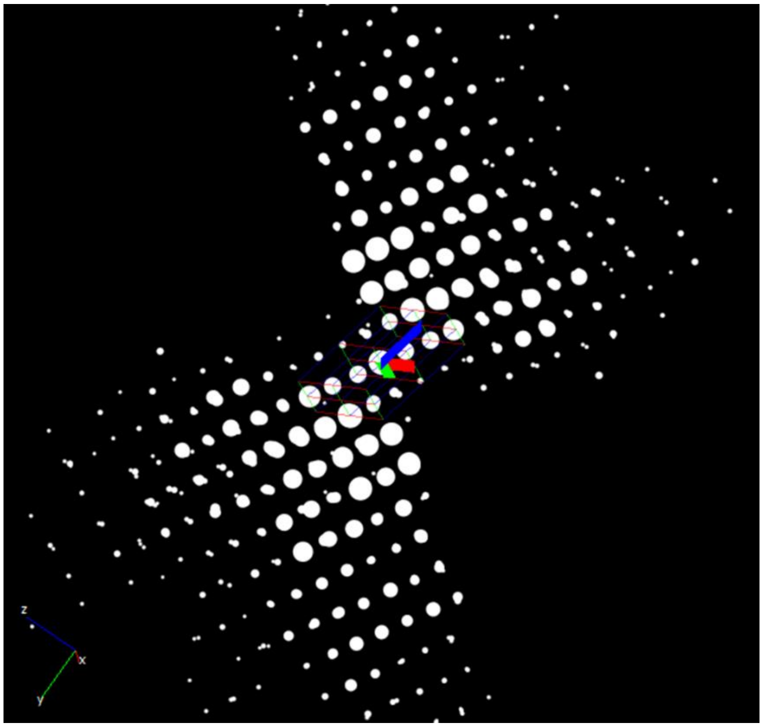

Supplementary Figure 20 | 3D reciprocal lattice of wCOF-Ag reconstructed from the SCED data. As wCOF-Ag was synthesized via post-synthesis, the local bonding is not very order. It is difficult to directly observe the newly formed bonds via SCED data.

Supplementary Table 2 | SCED: Experimental and Crystallographic parameters of wCOF-Ag.

Tilt range (°)	-53.22 ~ 50.17
Tilt step (°)	0.23
Wave length (Å)	0.0251
No. of frames	401
Exposure time per image (s)	0.5
Crystal system	Monoclinic
Possible space group	*C*2/*c*, *Cc*, *C*2/*m*, *Cm*, *C*2
Unit cell parameters	a = 15.74 Å, b = 31.11 Å, c = 11.00 Å, α = 90°, β = 122.6°, γ = 90°
Resolution (Å)	1.15
$I/\sigma(I)$	2.54
Completeness (%)	72.4
$CC_{1/2}$	95.6
R_{meas} (%)	27.2
No. of total reflections	2775
No. of unique reflections	1145

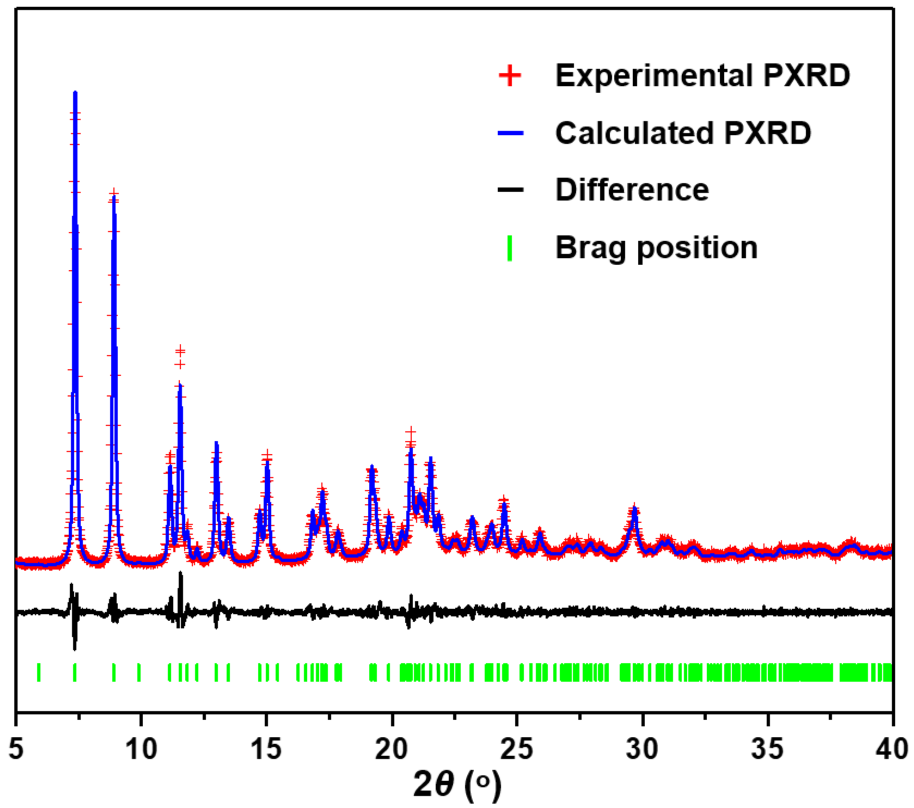

Supplementary Figure 21 | Pawley fitting of wCOF-Ag. Experimental (red), calculated (blue), as well as difference profiles (black) are presented. The position of Bragg reflections under the patterns are shown in green. The refined unit cell parameters are a = 15.6958 Å, b = 29.9200 Å, c = 10.6661 Å, and β = 123.31° (space group: $C2$), which are in good agreement with those of mCOF-Ag, indicating the parent structure of mCOF-Ag has been well inherited by wCOF-Ag. The final residuals for the Pawley fitting are R_{wp} = 0.135, R_{exp} = 0.123.

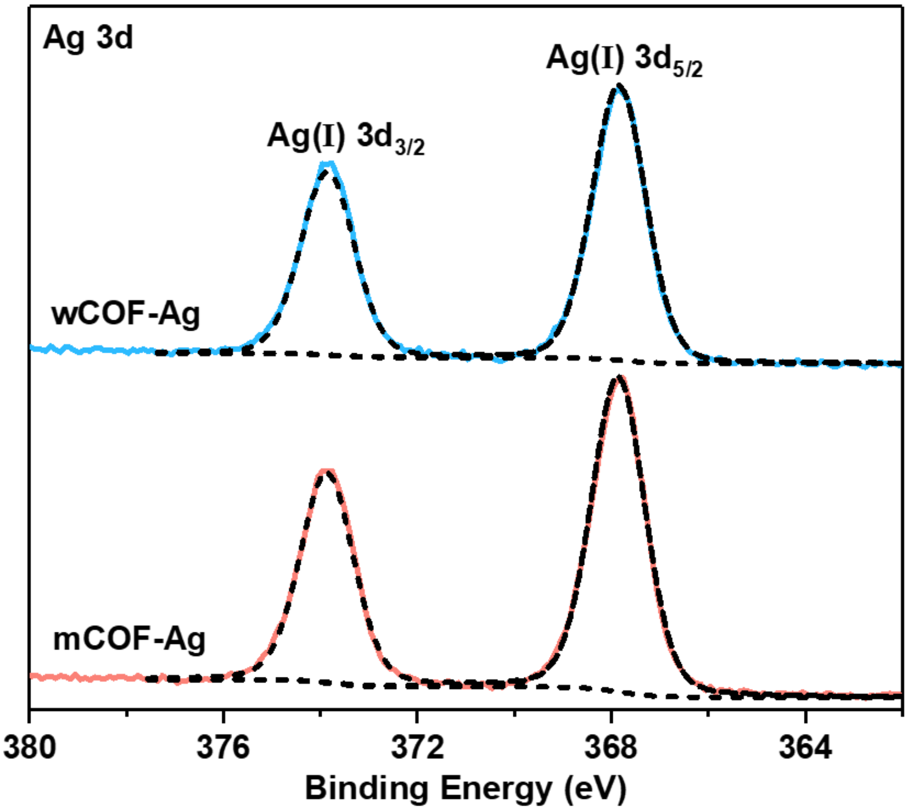

Supplementary Figure 22 | X-ray photoelectron spectroscopy (XPS) Ag3d data of mCOF-Ag and wCOF-Ag. The XPS Ag3d spectrum of wCOF-Ag is nearly identical to that of mCOF-Ag, indicating that Ag ions in both materials have similar chemical environments.

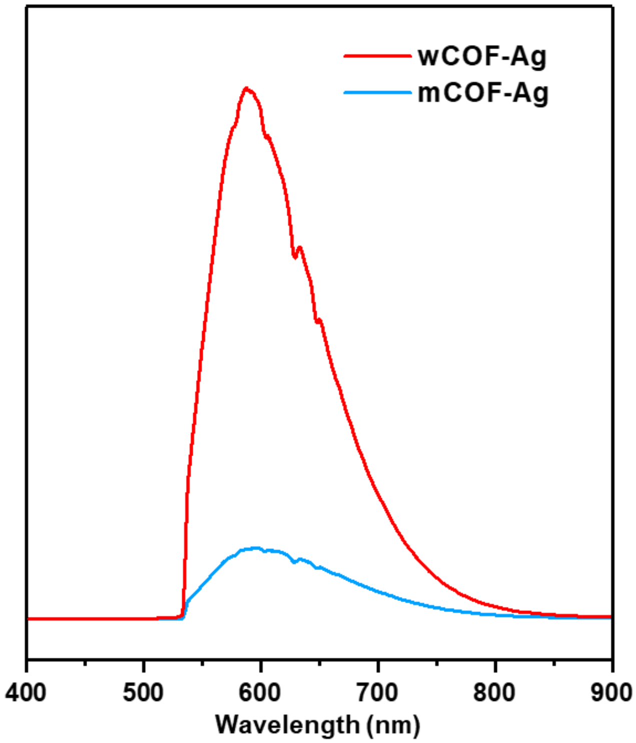

Supplementary Figure 23 | PL spectra of mCOF-Ag (before crosslinking) and wCOF-Ag (after crosslinking) excited by 532 nm laser.

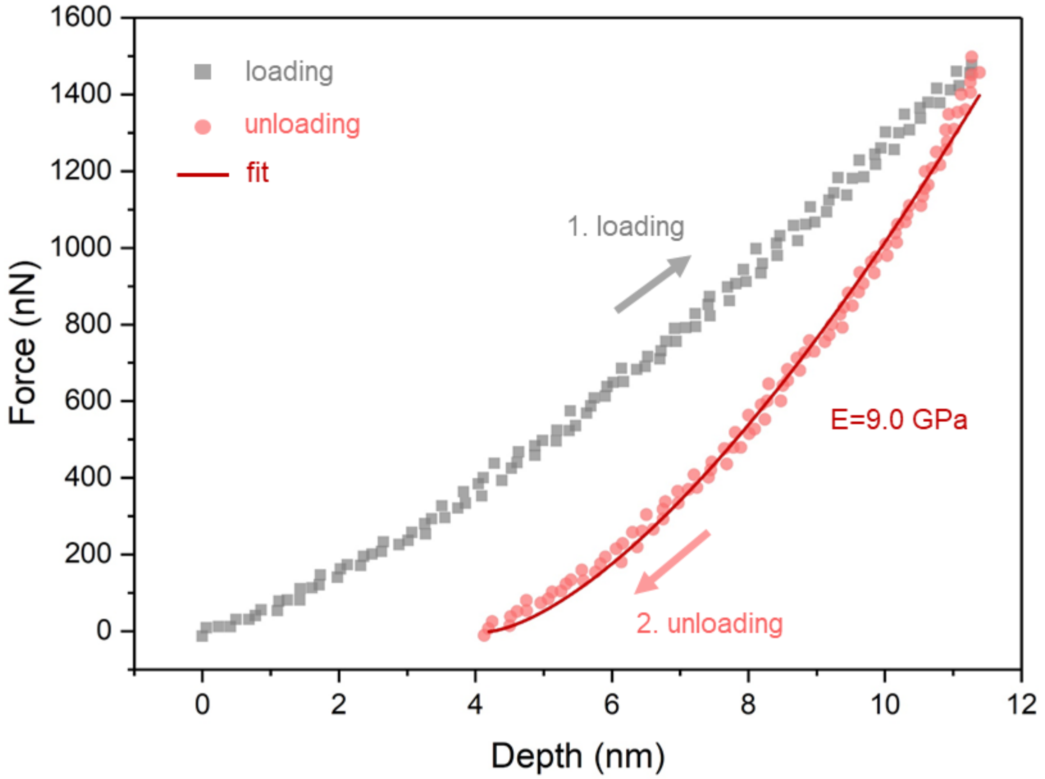

Supplementary Figure 24 | AFM nano-indentation force−displacement curve of an isolated crystal of mCOF-Ag. Following the standard analysis of nano-indentation, Young's modulus is related to the indentation curve by Equation 1: Hertzian model $F = \frac{4}{3}\frac{E}{(1-v^2)}\sqrt{R}\delta^{3/2}$, where F = force (from force curve), E = Young's modulus (fit parameter), ν = Poisson's ratio (sample dependent, 0.3), R = radius of the indenter (30.0 nm), δ = indentation.

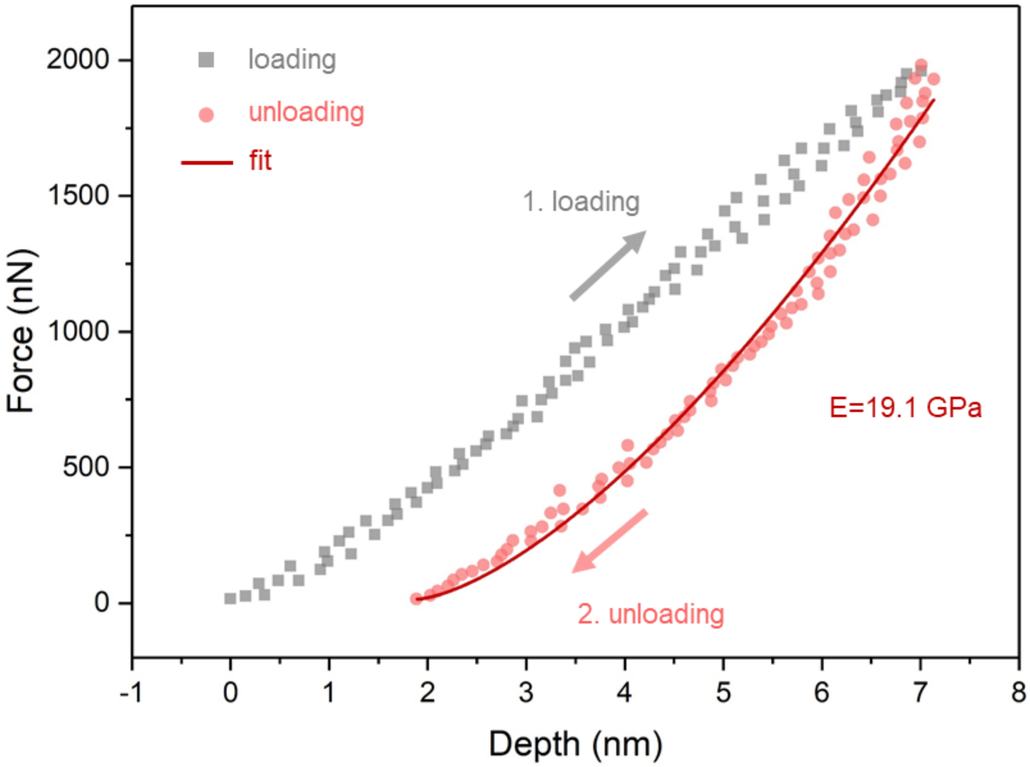

Supplementary Figure 25 | AFM nano-indentation force−displacement curve of an isolated crystal of wCOF-Ag. Following the standard analysis of nano-indentation, Young's modulus is related to the indentation curve by Equation 1: Hertzian model $F = \frac{4}{3}\frac{E}{(1-v^2)}\sqrt{R}\delta^{3/2}$, where F = force (from force curve), E = Young's modulus (fit parameter), ν = Poisson's ratio (sample dependent, 0.3), R = radius of the indenter (30.0 nm), δ = indentation.

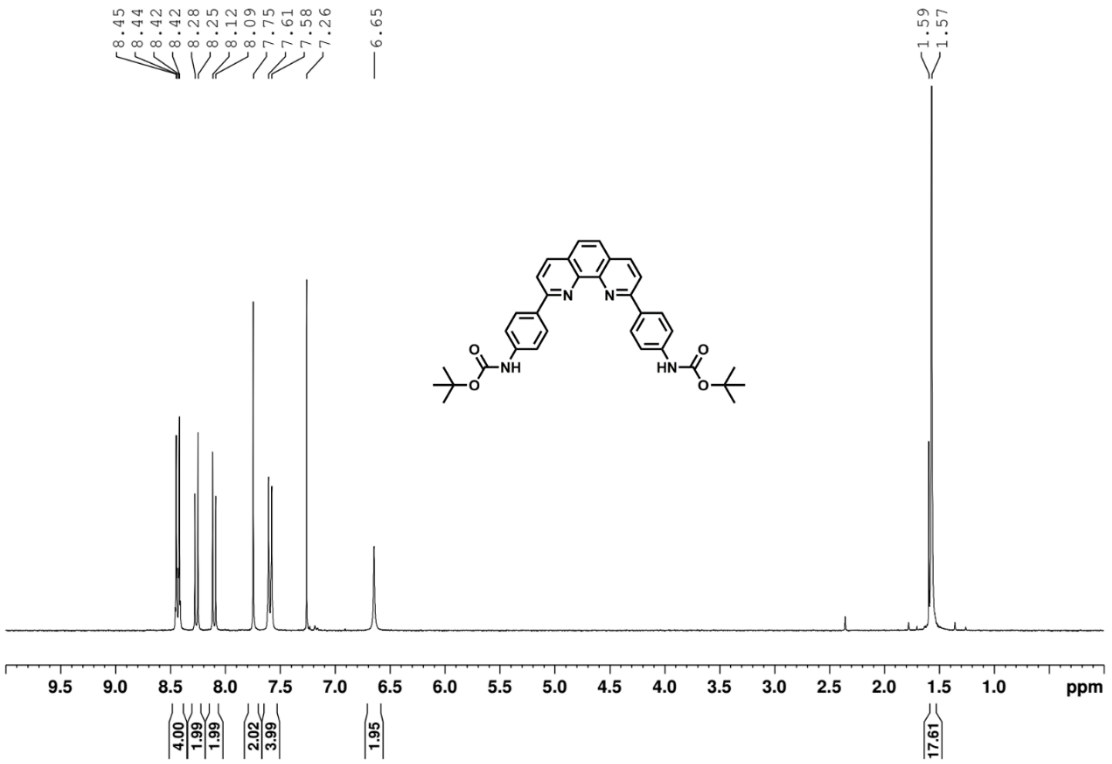

Supplementary Figure 26 | ^{1}H-NMR spectrum of compound **S2** (25 °C, 300 MHz, $CDCl_3$).

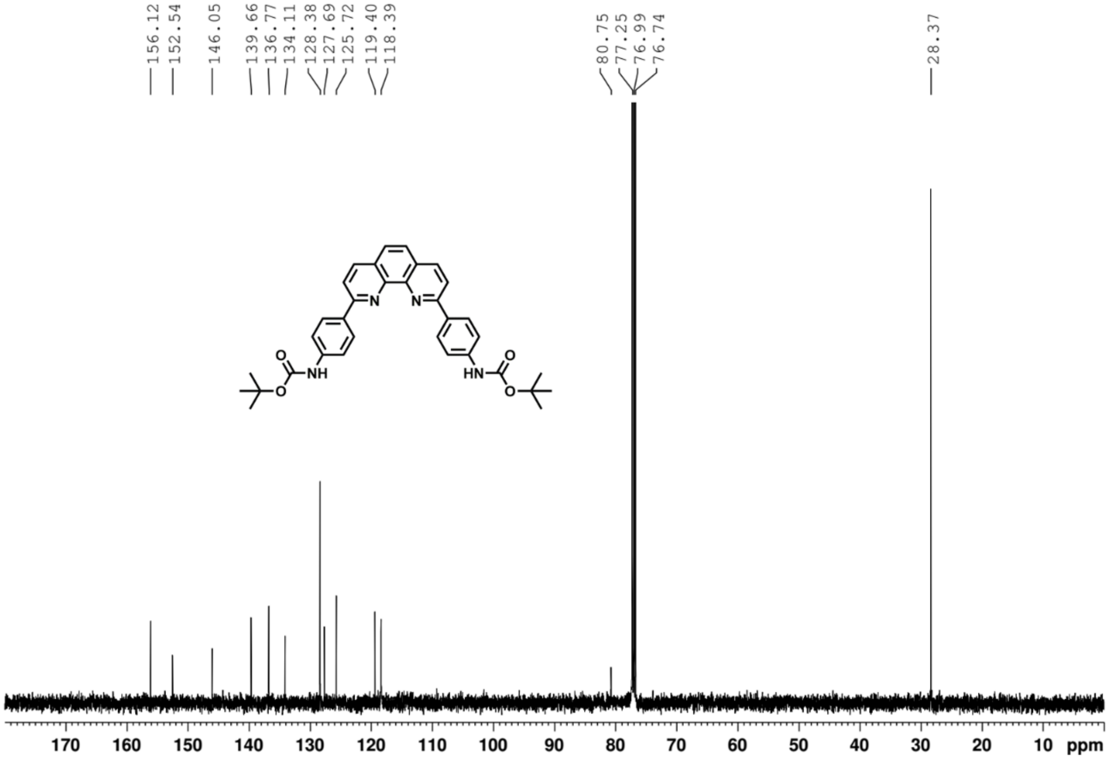

Supplementary Figure 27 | ^{13}C-NMR spectrum of compound **S2** (25 °C, 125 MHz, $CDCl_3$).

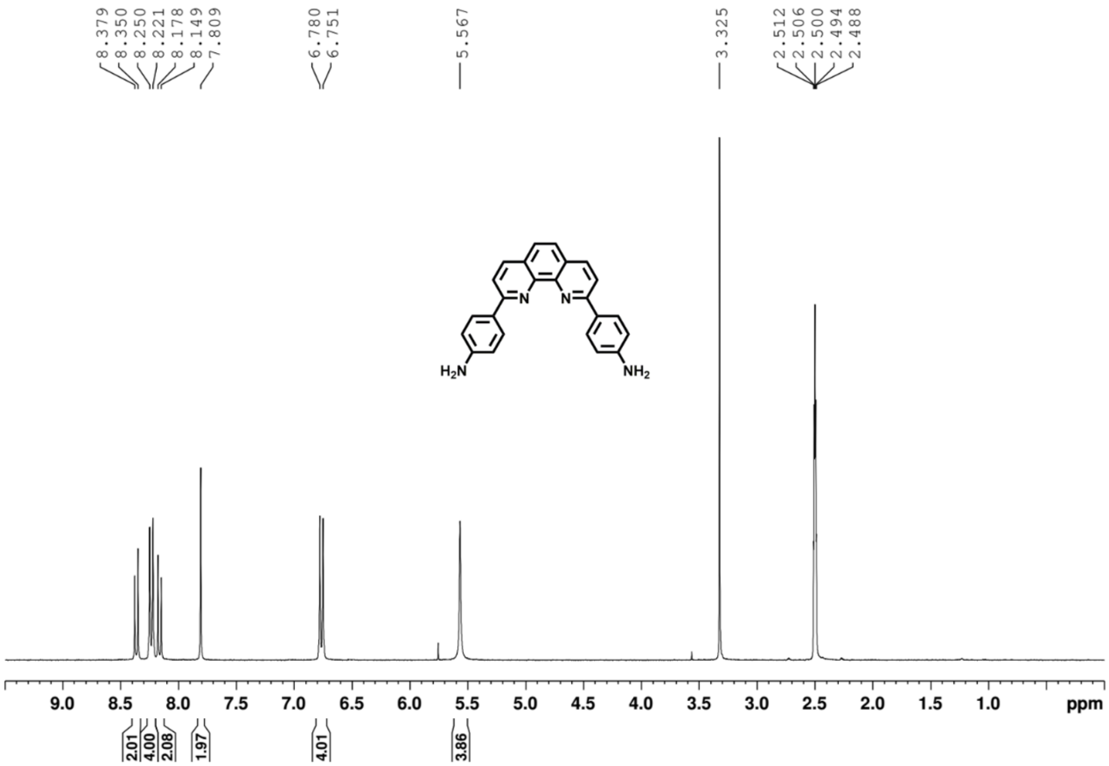

Supplementary Figure 28 | ^{1}H-NMR spectrum of **I** (25 °C, 300 MHz, DMSO-d_6).

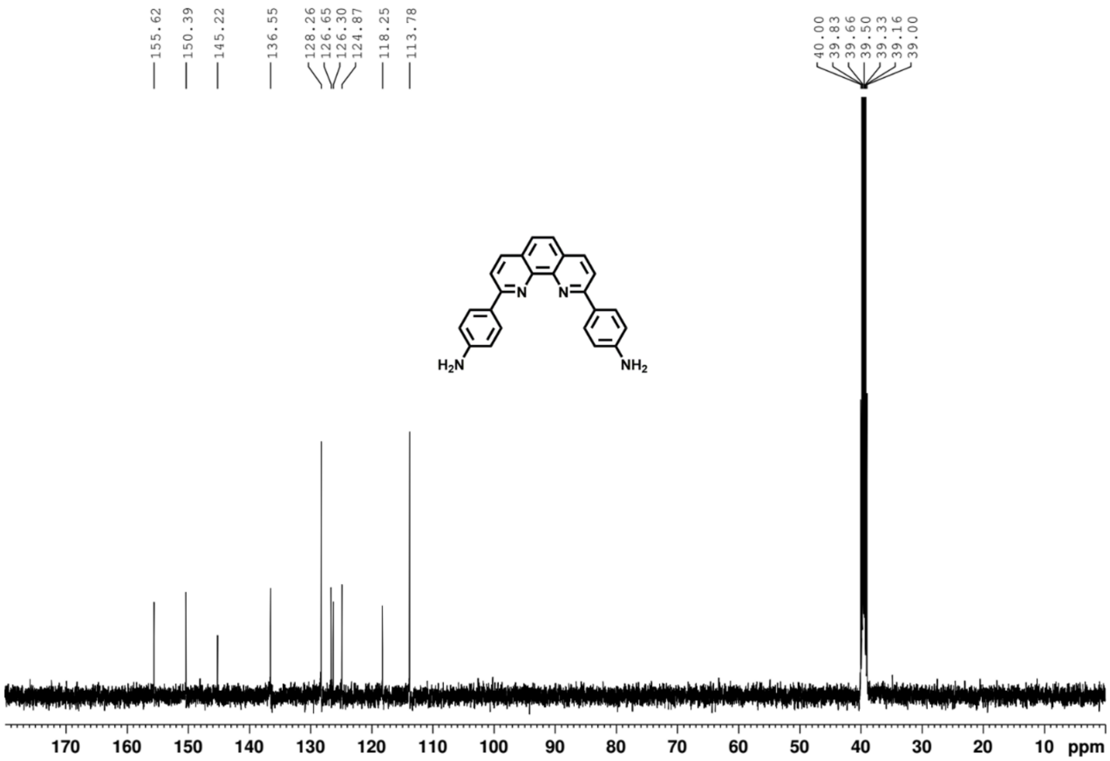

Supplementary Figure 29 | ^{13}C-NMR spectrum of **I** (25 °C, 125 MHz, DMSO-d_6).

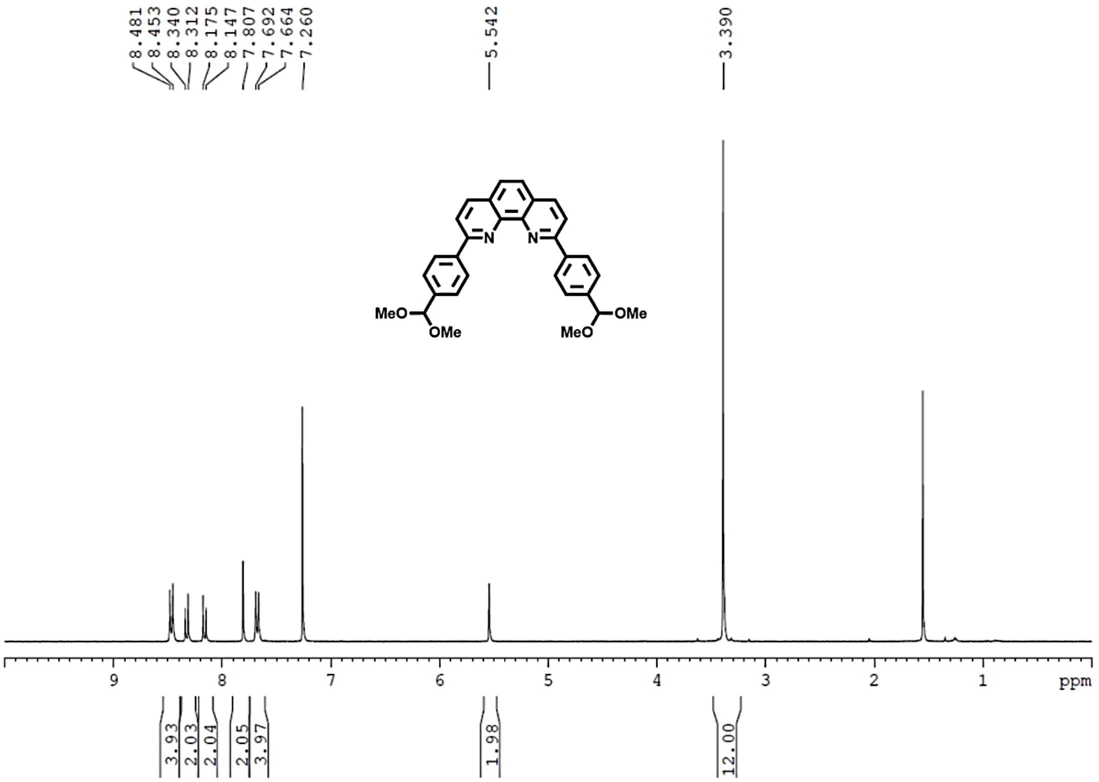

Supplementary Figure 30 | ^{1}H-NMR spectrum of **II** (25 °C, 300 MHz, $CDCl_3$).

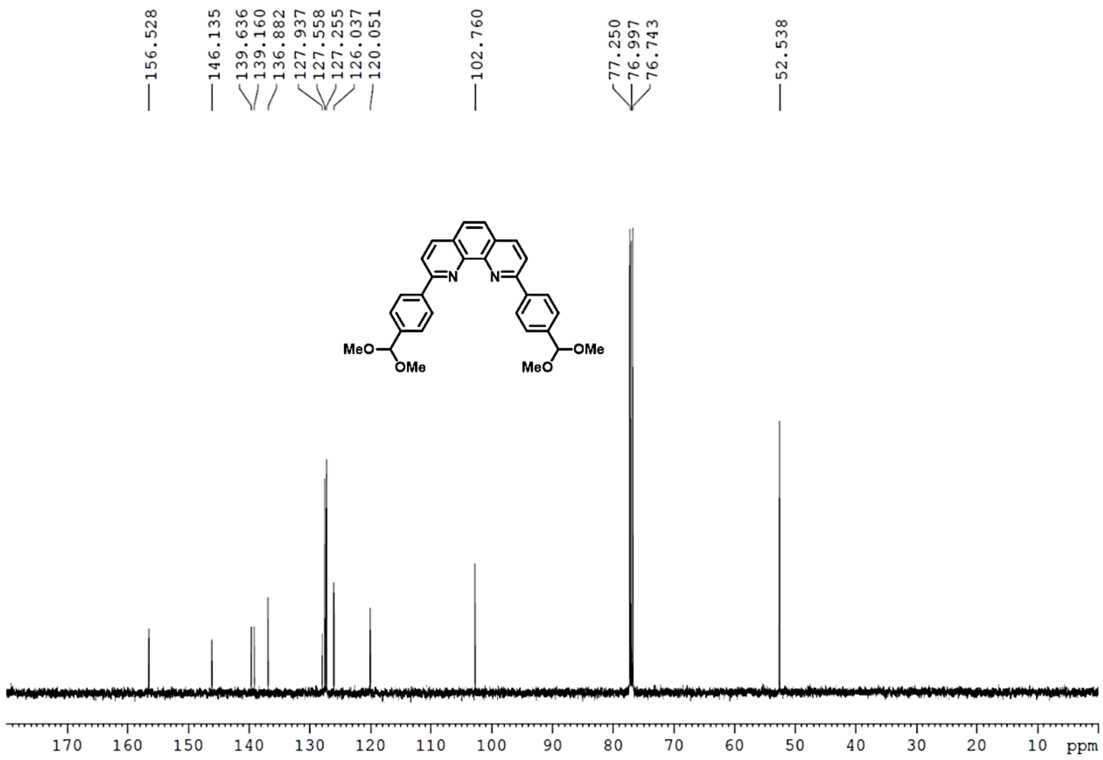

Supplementary Figure 31 | ^{13}C-NMR spectrum of **II** (25 °C, 125 MHz, $CDCl_3$).

Supplementary References:

1. Clary, J. W. *et al.* Hydride as a leaving group in the reaction of pinacolborane with halides under ambient Grignard and Barbier conditions. One-pot synthesis of alkyl, aryl, heteroaryl, vinyl, and allyl pinacolboronic esters. *J. Org. Chem.* **76**, 9602-9610 (2011).

2. Kabsch, W. XDS. *Acta Crystallogr. Sect. D.* **66**. 125-132 (2010).

3. Wan, W., Sun, J., Su, J., Hovmöller, S. & Zou, X. Three-dimensional rotation electron diffraction: software RED for automated data collection and data processing. *J. Appl. Crystallogr.* **46**, 1863-1873 (2013).
